# Molecular mechanisms of transcription factor mediated cell reprogramming: conversion of liver to pancreas

**DOI:** 10.1042/BST20200219

**Published:** 2021-03-05

**Authors:** Sebastian L. Wild, David Tosh

**Affiliations:** Department of Biology and Biochemistry, University of Bath, Bath BA2 7AY, U.K.

**Keywords:** liver, pancreas, reprogramming, transcription factor, transdifferentiation

## Abstract

Transdifferentiation is a type of cellular reprogramming involving the conversion of one differentiated cell type to another. This remarkable phenomenon holds enormous promise for the field of regenerative medicine. Over the last 20 years techniques used to reprogram cells to alternative identities have advanced dramatically. Cellular identity is determined by the transcriptional profile which comprises the subset of mRNAs, and therefore proteins, being expressed by a cell at a given point in time. A better understanding of the levers governing transcription factor activity benefits our ability to generate therapeutic cell types at will. One well-established example of transdifferentiation is the conversion of hepatocytes to pancreatic β-cells. This cell type conversion potentially represents a novel therapy in T1D treatment. The identification of key master regulator transcription factors (which distinguish one body part from another) during embryonic development has been central in developing transdifferentiation protocols. Pdx1 is one such example of a master regulator. Ectopic expression of vector-delivered transcription factors (particularly the triumvirate of Pdx1, Ngn3 and MafA) induces reprogramming through broad transcriptional remodelling. Increasingly, complimentary cell culture techniques, which recapitulate the developmental microenvironment, are employed to coax cells to adopt new identities by indirectly regulating transcription factor activity via intracellular signalling pathways. Both transcription factor-based reprogramming and directed differentiation approaches ultimately exploit transcription factors to influence cellular identity. Here, we explore the evolution of reprogramming and directed differentiation approaches within the context of hepatocyte to β-cell transdifferentiation focussing on how the introduction of new techniques has improved our ability to generate β-cells.

## Introduction

The history of cell reprogramming, or the manipulation of cellular identity, spans a century of technological and conceptual innovation across developmental biology, biochemistry and medicine (for reviews see [1,2]). Reprogramming techniques continue to evolve as we learn more about the developmental and molecular cues which govern cellular differentiation and identity. Here, we discuss how transcription factors, and their unique position as the gatekeepers of cellular identity, are exploited in cell reprogramming protocols by exploring work focusing on one reprogramming paradigm — the transdifferentiation of hepatocytes to pancreatic beta cells (β-cell). We consider how, over the last 20 years, advances in genetic engineering and cell culture techniques have improved the efficiency and efficacy of the transdifferentiation process and brought us closer to a clinically relevant therapy for type 1 diabetes (T1D). We examine how transdifferentiation protocols are evolving to ever more faithfully recapitulate normal developmental biology using increasingly sophisticated biomimetic techniques and ectopic transcription factor expression.

Cellular reprogramming refers to the erasure of epigenetic markers allowing for the remodelling of chromatin, gene expression and ultimately cell identity. More specifically, cell reprogramming can be defined as the process of effecting stable change to cellular identity through revision of the transcriptional profile (the complement of mRNA transcribed by a cell at a given point in time) via manipulation of epigenetic modifiers. Reprogramming necessarily involves a switch from expression of one set of genes to another, where each gene set represents a distinct cellular identity.

**Figure 1.**
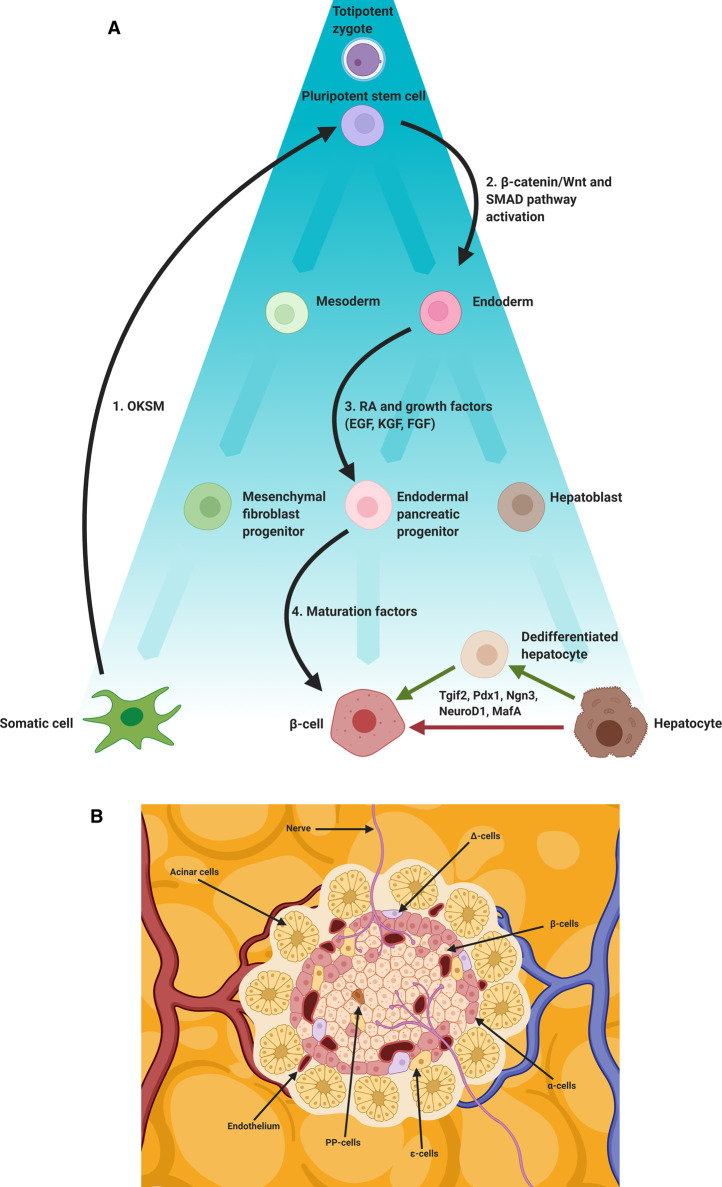
β-cell programming and reprogramming. (**A**) Schematic representation of embryological differentiation from zygote to differentiated somatic cell (β-cell, hepatocyte), the directed differentiation of OKSM iPSCs to β-cell and the transdifferentiation of hepatocytes to β-cell using pancreatic master regulator transcription factors (Tgif2, Pdx1, Ngn3, NeuroD1, MafA). Black arrows indicate steps involved in the generation and subsequent programming of iPSCs; (**1**) Somatic cells transfected with OKSM transcription factors are reprogrammed to a pluripotent stem cell state using Yamanaka factors (OKSM), (**2**) the resulting iPSC can then be treated with small molecules, cytokines, morphogens and growth factors. Fate is directed toward an endoderm lineage, typically through activation of the β-catenin/Wnt and SMAD signalling pathways (**3**). Pancreatic progenitors are then induced, typically using retinoic acid (RA) in combination with a range of growth factors (EGF, KGF, FGF). Finally, (**4**) a β-cell phenotype is induced using various maturation factors. A recent study used a cocktail including Anaplastic Lymphoma Kinase Inhibitor II (ALKi), Triiodo-L-Thyronine (T3), PI3-K Inhibitor (XXI), ALK inhibitor LDN193189 (LDN) and vitamin C (Vc). Ectopic expression of pancreatic transcription factors (Pdx1, MafA, Ngn3, Tgif2, NeuroD1) can induce or contribute to transdifferentiation to a β-cell phenotype. Direct (no intermediary stage) and indirect (a dedifferentiated intermediary stage) routes are depicted. (**B**) β-cells reside in the islets of Langerhans and are subject to a specific niche involving mechanical and chemical cues. Blood borne factors (including oxygen (O_2_)), extra cellular matrix (ECM) components, neighbouring acinar, endocrine (α-cells, δ-cells, PP-cells, ε-cells), endothelial and neuronal cells all contribute to this niche.

Yamanaka and Takahashi's seminal 2006 work inducing pluripotency in differentiated cells to create the now famous induced pluripotent stem cell (iPSC) and Gurdon's work on somatic cell nuclear transfer (SCNT) are examples of cellular reprogramming [3,4]. In these classical cases, cellular identity is regressed to a multipotent state in response to exogenous pioneer transcription factors. In the case of Yamanaka and Takahashi these were Oct4, Klf4, Sox2 and c-Myc (OKSM) [4]. Pioneer transcription factors are associated with the opening of chromatin, permitting access to hitherto inaccessible regions of the genome by transcription factors [5,6]. Another category of cellular reprogramming refers to the conversion of one differentiated cell type to another differentiated cell type, sometimes referred to as direct cellular reprogramming or transdifferentiation. Transdifferentiation is defined as the stable conversion of one differentiated cell type to another, without the requirement for an intermediate step in which a cell is regressed to a multipotent state (Figure 1) [7]. Transdifferentiation, does not require the administration of pioneer factors *per se.* Rather, protocols typically rely on master regulator transcription factors to initiate a transcription cascade which likely involves the recruitment of endogenous pioneer factors in the reprogramming process. For example, the pancreatic pro-endocrine master regulator Neurogenin 3 (Ngn3) has been demonstrated to cooperate with the hepatic and pancreatic pioneer transcription factor Forkhead Box Protein A2 (FoxA2) to facilitate autoinduction [8]. Studies examining the interconversion of hepatocytes and β-cells have revealed that the down-regulation of genes associated with the starting cell type is required in order for genes associated with the desired cell type to be expressed [9,10]. While master regulator and pioneer transcription factors act as the catalysts of change in the production of iPSCs, in SCNT and in transdifferentiation their impact is ultimately maintained by the remodelling of chromatin by epigenetic modifiers including histone deacetylases and histone methyltransferases (for review see [11,12]). In this mini-review we will limit our discussion to the instructive role of pancreatic master regulators in liver to pancreas transdifferentiation and how the recapitulation of the *in vivo* environment using cell culture techniques can augment their impact in the generation β-cells.

## Transdifferentiation of liver to pancreas

Transdifferentiation has been demonstrated between several cell types and across germ layers including fibroblast to neuron [13], fibroblast to cardiomyocyte [14], pancreatic cell to hepatocyte [9] and fibroblast to hepatocyte [15]. The transdifferentiation of hepatocytes to β-cells represents one of the most clinically exciting cell type conversions due to the potential impact on society of its successful translation to therapy [14]. β-cells are the only physiologically relevant Insulin producing cells in the body. Rare spontaneously arising Insulin secreting cells have been reported in the biliary epithelium [16]. Though these are not considered a significant source of Insulin they might constitute an alternative source if they could be expanded [17]. T1D is caused by the autoimmune destruction of β-cells in the pancreas [18]. In adult pancreas, β-cells are found in the islets of Langerhans alongside other endocrine cell types; Glucagon secreting alpha (α)-cells, Somatostatin secreting delta (δ)-cells, Pancreatic Polypepetide (PP) secreting PP-cells and Ghrelin secreting epsilon (ε)-cells. Today Insulin therapy remains the frontline treatment for most T1D patients. Despite the effectiveness of modern formulations [19,20], challenges around maintaining blood glucose levels within the healthy range (4.0 mmol/L to 5.4 mmol/L during fasting) over a lifetime means diabetic complications are commonplace. The resultant dysregulation of glucose homeostasis has consequences for health including diabetic retinopathy, nephropathy and neuropathy. Replacing those lost and exquisitely adapted β-cells is therefore the subject of intense research with several cellular candidates being explored including stem cells [21], pancreatic exocrine cells [21,22] and hepatocytes, the primary parenchymal cell of the liver. Proponents of the latter approach speak of the shared developmental lineage of liver and pancreas; both being derived from the foregut endoderm [23]. Furthermore, both hepatocytes and β-cells rely on a common glucose sensing machinery. The components of which include the Glucose Transporter 2 (GLUT2) [24] and the hepatic hexokinase enzyme Glucokinase [25]. Several transcription factors are also common to both cell types including hepatocyte nuclear family (Hnf) members Hnf1α, Hnf4α and Hnf6 [26], Haematopoietically Expressed Homeobox (Hhex) [27,28] and the pioneer transcription GATA Binding Protein 4 (GATA4) [29]. Moreover, the liver possesses the remarkable ability to regenerate thereby representing a potentially replenishable source of cells for transdifferentiation [30].

## Reprogramming strategies

The interconversion of hepatocytes and another type of pancreatic cell — the acinar cell, can occur in response to carcinogens [31] and in liver cirrhosis [16,32]. Examples of an experimental pancreatic cell to hepatocyte transdifferentiation include the conversion of the rat pancreatic cell line AR42J-B13 to a hepatocyte phenotype in response to treatment with the synthetic glucocorticoid dexamethasone [9] and through feeding rats a copper depleted diet in combination with a copper chelator [33,34]. Dexamethasone acts to repress the pancreatic phenotype and induce the hepatic phenotype through induction of the key regulatory transcription factor CCAAT-Enhancer-Binding Protein Beta (C/EBPβ) [9,35]. When Pancreatic Duodenal Homeobox 1 (Pdx1) is expressed in hepatocytes it suppresses C/EBPβ to promote the pancreatic phenotype [10]. Suppression of hepatic genes is now recognised as a critical step in the transdifferentiation process. More recently the TALE protein transcription factor TGFβ Induced Factor 2 (Tgif2) has been shown to similarly suppress the hepatic phenotype and induce a pancreatic progenitor phenotype [36].

The transdifferentiation of hepatocytes to a β-cell like phenotype has been demonstrated with several transcription factors alone and in combination in various models including mouse [37], *Xenopus* [38], primary human cells [39] and a human hepatoma cell line [40]. Reprogramming methods are typically centred around the administration of ectopic transcription factors, such as in liver to pancreas transdifferentiation, and in the generation of induced pluripotent stem cells using the Yamanaka (OKSM) factors [4]. The reprogramming process is subsequently fine-tuned through manipulation of the microenvironment to direct differentiation to a desired phenotype [21]. Expression of ectopic transcription factors in the case of iPSCs, initiates broad transcriptional remodelling with multiple downstream signalling cascades effected. Microenvironment manipulation involves the use of small molecules and other environmental cues to act as triggers for activating intracellular pathways and upstream effectors (likely master regulatory transcription factors). Both approaches ultimately remodel transcriptional profiles through transcription factor activity. Figure 1 highlights differences in reprogramming to β cell approaches and endogenous and exogenous cues involved in their programming.

## Ectopic expression of master regulatory transcription factors to induce transdifferentiation

A master regulator transcription factor is, according to coiner Susumu Ohno, a ‘gene that occupies the very top of a regulatory hierarchy,’ which, ‘by its very definition should not be under the regulatory influence of any other gene’ [41]. While many genes considered to be master regulators are regulated in some way by other genes, they are generally homeotic and thus responsible for initiating the development of specific organs or tissues. Their relatively high position in the transcriptional hierarchy means they produce attributes considered critical in defining a particular cell type. Chan and Kyba recently attempted to update the definition of a master regulator to accommodate new science [42]. They imagined a gene that is (i) expressed at the inception of a developmental lineage, (ii) participates in the specification of that lineage by regulating multiple downstream genes and (iii) specifies the fate of cells when mis-expressed [42]. In terms of Chan and Kyba's criteria, the pancreatic master transcription factor Pdx1 is expressed in the foregut endoderm of the developing embryo and is required for the development of the pancreatic bud [43,44]. Pdx1 is also critical to the maintenance of adult β-cells through binding of TAAT motifs in the promoters of downstream pancreatic transcription factors including Hnf1β, FoxA2, Ngn3 and MafBZIP Transcription Factor A (MafA) [45–47]. Critically, mice lacking Pdx1 expression exhibit pancreatic agenesis demonstrating a key role in pancreatic development [48].

In a landmark study in 2000 Sarah Ferber and colleagues reported that the reprogramming of mouse hepatocytes to a β-cell lineage could be achieved simply via ectopic expression of adenoviral-mediated delivery of Pdx1 [37]. Ectopic Pdx1 expression induced expression of the β-cell specific Insulin genes INS1 and INS2 as well as Prohormone Convertase (PC1/3), a protease that cleaves Proinsulin to produce mature Insulin and C-peptide [49]. Furthermore, cells were capable of glucose-stimulated insulin secretion (GSIS), a cardinal feature of β-cell function, and ameliorated hyperglycaemia in a streptozotocin (STZ) model of diabetes in mice. STZ is a β-cell specific toxin used to model T1D. Despite this success, levels of mature Insulin were ∼18 fold lower in the livers of mice exposed to adenovirus expressing Pdx1 when compared with control pancreas [37]. Subsequent experiments revealed that the endocrine markers glucagon and somatostatin, the exocrine pancreatic marker amylase and the exocrine transcription factor p48 were also induced in response to Pdx1 expression in the liver of both mouse and *Xenopus* [38,50]. These early experiments relied solely on an *in vivo* transcription factor-based approach. Though promising, several hurdles hinder the translation to bedside therapy including overcoming the risk of inflammatory responses triggered by viral vectors [51]. Furthermore, ectopic expression of pancreatic genes have been shown to cause hepatic dysmorphogenesis [52] and fulminant hepatitis [53]. Interestingly and somewhat counterintuitively, these same immune responses have been shown to contribute to the efficiency of transdifferentiation *in vivo* [54]. This possibly reflects the importance of active Wnt signalling in conferring transdifferentiation propensity [55] as Wnt/β-catenin signalling is known to be up-regulated in hepatocytes across the lobule in response to injury [56].

Ectopic expression of master regulatory transcription factors is still critical to our ability to transdifferentiate from liver to pancreas [57]. Candidate transcription factors (Pdx1, Ngn3, MafA, Tgif2) have been targeted due to having been identified as important in normal pancreatic development [36,48,58]. However, screening candidates is a time consuming and costly process and differences in activity *in vitro* further complicates the process. Bioinformatic approaches have been applied to this problem [59,60]. One such tool (Mogrify) combines gene expression data (FANTOM5 [61]) with regulatory network data to predict transcription factors that might be instructive in converting cell types. By identifying which transcription factors are most responsible for transcriptional profiles associated with cell type researchers successfully predicted transcription factors confirmed to induce reprogramming such as the use of MyoD1 in the conversion of fibroblasts to myoblast and OKSM factors in the production of iPSCs from fibroblasts [62].

## Polycistronic and hierarchical transcription factors

Following the success of early *in vivo* experiments [37,38,50] efforts focused on producing a mature β-cell phenotype that could meet the demands of glucose metabolism both in terms of capacity (insulin production) and reliability i.e. GSIS in response to repeated glucose challenges. Experiments in *Xenopus* using the Pdx1 orthologue XlHbox8 found success when combined with a constitutive transcriptional activator VP16 transcript [38]. This raised questions around the requirement of co-activators to enable expression of Pdx1 in ectopic tissues where such co-activators might be absent. Conversely, using transcriptional repressors for a particular cell phenotype in combination with ectopic expression of transcription factors might aid in enhancing the reprogramming process.

Researchers hypothesised that expression of transcription factors downstream of Pdx1, known to be present in maturing β-cells, might more closely recapitulate normal development. Taking a combinatorial approach might therefore be more effective at reprogramming. In 2003 the first study to utilise this approach investigated the effects of Neuronal Differentiation Factor 1 (NeuroD1), a basic helix–loop–helix (bHLH) transcription factor family member downstream of Pdx1 known to drive Insulin production in complex with Pdx1 and an EGF family member Betacellulin (Btc), an EGF receptor ligand [63,64]. The combined expression of NeuroD1 and Btc was found to ameliorate hyperglycaemia in an STZ model with the combination outperforming NeuroD1 or Btc alone. Pdx1 expression however was found to drive fulminant hepatitis, possibly as a result of the broad and sustained expression of Pdx1 throughout the liver. This overexpression of Pdx1 repressed the hepatic phenotype causing fulminant hepatitis and hepatic agenesis [53]. This effect was not observed with NeuroD1 or Ngn3 alone [10]. NeuroD1/Btc induced expression of upstream and downstream genes including Pdx1, Ngn3, Pax4, Pax6, Nkx2.2, Nkx6.1 and Isl-1 and corroborated Ber's observation of autoinduction, a finding later confirmed by Banga et al. [65].

Ngn3 is a bHLH transcription factor downstream of Pdx1 which promotes exit from the cell cycle and is critical for specification and differentiation of endocrine pancreas [2,66]. Transient expression of Ngn3 occurs during the secondary transition (between mouse embryonic day (E)12.5–E15.5) of pancreatic development and functions as a gatekeeper for endocrine fated cells [67]. Ablation of Ngn3 results in a failure to produce endocrine cells [58]. In 2009 the Chan lab [68] used ectopic Ngn3 in combination with Btc to induce production of Insulin *in vivo* in the liver of a murine STZ model. Conversion towards a β-cell phenotype was observed in two distinct cell populations — hepatocytes and putative bipotent progenitors (termed ‘oval’ cells). There was a transient lineage switch in hepatocytes whereas oval cells exhibited a more prolonged lineage switch. This difference in potential perhaps reflects an insufficiency of transcription factors downstream of Pdx1 to bring about a stable change in phenotype. Long term reprogramming had previously been noted following transient expression using Pdx1 [50].

The combination of Pdx1, Ngn3 and MafA converts pancreatic exocrine tissue to an endocrine phenotype [69]. MafA is a transcription factor known to be critical to the maturation of β-cells [46]. The same combination *in vivo* in mouse liver converted a Sox9 positive sub-population of hepatocytes to a β-cell phenotype [65]. Though suggested as likely ductular in origin, Sox9 has subsequently been reported to be expressed at low levels in some periportal hepatocytes (representing ∼4.53% of the total hepatocyte population) [70]. This is in conflict with the perivenous transdifferentiation pattern seen in many studies [55].

Important to normal development is the hierarchical and temporal context within which transcription factors are expressed [46,58]. Berneman-Zeitouni and colleagues conducted a screen of sequentially expressed combinations of Pdx1, Pax4 and MafA. They found that the order in which genes are expressed influences maturation with the Pdx1–Pax4–MafA producing the highest number of mature phenotype β-cells determined by GSIS and production of mature Insulin [71].

Tgif2 is expressed in the definitive endoderm progenitor pool where its expression is subsequently up-regulated in pancreatic progenitors and down-regulated in hepatic progenitors [72,73]. Cerda-Esteban and colleagues induced transdifferentiation of hepatocytes to a β-cell phenotype through ectopic expression of Tgif2 resulting in expression of a range of pancreatic genes including Pdx1 [36]. Ma and colleagues recently tested the sequential lipid-based transfection of Tgif2, Pdx1, NeuroD1 and MafA mRNAs in primary mouse hepatocytes over four days. The authors compared results from protocols expressing Tgif2 alone and different combinations of Pdx1, NeuroD1 and MafA [36]. They reported significant increases in Insulin production and GSIS in particular when transcription factors were expressed sequentially and hierarchically. The authors suggest the increased efficiency was in part due to increased plasticity in response to up-regulation of various Wnt signalling pathway associated genes including the Wnt/Planar-Cell-Polarity pathway (PCP) genes VANGL and CELSR as well the β-catenin dependent canonical Wnt signalling pathway suppressor Tle3. Interestingly Tcf7, a β-catenin co-activator and Jnk (a protein downstream of the Wnt/PCP signalling pathway) were found to be up-regulated in transfected cells. The authors suggest that remodelling of cell polarity may have contributed to transdifferentiation efficiency. Given the known role of the canonical Wnt pathway in liver to pancreas transdifferentiation [55] and known positive regulation of canonical Wnt by Tgif2 [74] as well as the conflicting data with respect to Wnt pathway activation, it is possible that up-regulation of β-catenin dependent transcription endowed these cells with increased plasticity.

## Soluble factors and directed differentiation *in vitro*

In 2005 Sapir and colleagues were the first to demonstrate the *in vitro* transdifferentiation of human hepatocytes to a β-cell phenotype [39]. *In vitro* reprogramming presents new challenges as the extracellular milieu containing endogenous survival factors is lacking. However, it does allow for greater control of the microenvironment and identification of dependent variables. Hepatocytes were cultured with EGF and soluble vitamin B3 (nicotinamide). Each soluble factor had previously been shown to promote the β-cell phenotype [75,76]. The effects of Pdx1 transfection were compared alone and in combination with soluble factors and revealed that Insulin production in transdifferentiated β-cells increased by two orders of magnitude when co-treated with EGF and nicotinamide [39]. Furthermore, the cells were capable of GSIS as well as containing dense core Insulin granules and the capacity to ameliorate an STZ model of diabetes when transplanted into mice comparable with previous *in vivo* transdifferentiation attempts [50]. By demonstrating the utility of soluble factors and of autologous transplantation of *ex vivo* generated β-cells the authors paved the way for future *ex vivo* protocols [39]. Later, the same group demonstrated that a Glucagon-Like Peptide (GLP) receptor agonist Exendin-4 similarly enhanced the efficiency of the transdifferentiation process as well as maturation of β-cells [77].

Recently, Motoyama et al., screened a number of soluble factors in an effort to further improve the efficiency of liver to pancreas transdifferentiation. This approach had been relatively underexplored within the context of liver to pancreas transdifferentiation whilst extensively explored in stem cell differentiation protocols [21,78–80]. Administration of eight soluble factors (N2 (human Transferrin, human Insulin, Progesterone, Putrescine, sodium selenite), Exendin-4, L0685,485 (a Notch inhibitor) and Noggin (a TGFβ inhibitor) in mouse bipotent hepatic progenitor cells promoted β-cell maturation, increasing intracellular C-peptide by 12-fold and INS gene transcription 1.5–2 fold when compared with β-cells produced using transcription factors alone [78].

The canonical Wnt/β-catenin pathway activity in perivenous hepatocytes confers an increased capability to transdifferentiate to a β-cell phenotype when compared with hepatocytes of the mid-lobular or periportal regions [55]. The canonical Wnt/β-catenin pathway is known to be involved in the homeostatic renewal and regeneration in response to injury across the liver [56]. Cohen et al. hypothesised that gene activity, downstream of Wnt signalling, serves to model chromatin in such a way as to confer plasticity in perivenous hepatocytes and restrict plasticity in Wnt inactive hepatocytes. They utilised the histone deacetylase inhibitors (HDACi) suberoylanilide hydroxamic acid and sodium butyrate to make available Wnt target genes and pancreatic genes through chromatin opening that would otherwise be repressed. An increase in the transdifferentiation efficiency in HDACi treated cells in response to sequential adenovirus delivered Pdx1, MafA and NeuroD1 was observed [55].

## The 3D environment

The maintenance of cellular identity is contingent on the presence and integration of numerous factors [81,82]. The cellular phenotype may in part be determined by cell–cell interactions and through interactions with components of the ECM. Molecular and mechanical signals generated by the physical environment influence transcription of genes and thus help to determine cellular identity. This chorus of signals evolves synergistically with the developing embryo. Differentiating cells express and secrete different ECM proteins (proteoglycans, glycoproteins, laminins and collagens) at different times during development. ECMs are therefore at once a product and determinant of cell type. In the pancreas, these signals guide differentiation from pancreatic precursors to β-cell [81]. Within the context of liver-pancreas transdifferentiation the role of the 3D environment has been underexplored. Studies in the directed differentiation of stem cells routinely use cell clustering and 3D culture techniques to enhance the maturation of iPSCs to a β-cell phenotype [21,79,80]. Building on the success of their iPSC to β-cell differentiation regime [79] the Hebrok lab explored how recapitulation of the *in vivo* situation may promote maturation *in vitro*. Hypothesising that cell reorganisation and endocrine cell clustering are key features driving pancreatic development they added a β-cell enrichment step prior to cell clustering. scRNA-seq and gene set enrichment analysis revealed enhanced expression of metabolically mature β-cell genes associated with oxidative metabolic pathways including oxidative phosphorylation, electron transport chain, TCA cycle and ATP biosynthesis when compared with non-enriched clusters [21]. A recent study examined the ability of transdifferentiated β-cells to form clusters on a 3D gelatin culture medium though ability to form clusters was used as a measure of phenotype rather than as a treatment [57].

Proteomic analyses have compared hepatocytes cultured in 3D spheroid culture and ‘sandwich’ culture (where cells are cultured between two layers of ECM (in this case a Collagen I substratum overlaid with a Matrigel gel matrix) with traditional monolayer culture on an ECM substratum. 3D culture was superior in maintaining the hepatic phenotype, while sandwich culture was superior to monolayer culture [84]. *In vitro* liver-pancreas transdifferentiation studies have occasionally made use of ECMs in monolayer culture notably fibronectin [39] and collagen 1 [36] while others used no ECM [57,78]. Avoiding ECM components that might maintain a hepatic phenotype may benefit transdifferentiation protocols through promoting a degree of dedifferentiation and plasticity. It is also important to consider attempts to recapitulate the ECM environment of the pancreas.

## O_2_

O_2_ is essential to the survival of the developing embryo. Beyond its role in ATP production, low O_2_ tension (PO_2_), or hypoxia, is sensed by cells via the pleiotropic hypoxia-inducible factor (HIF) pathway. Low PO₂ in early embryonic pancreas inhibits proliferation in embryonic stem cells through regulation of cyclin-dependent kinases inhibitors p21 and p27 [82,85,86]. Hypoxia permits the cytoplasmic dimerisation of bHLH HIF alpha subunits (HIF1α, HIF2α, HIF3α) with constitutively expressed bHLH beta-subunits. The heterodimers subsequently translocate to the nucleus where they complex with CBP/p300 to bind HIF response elements within gene promotors to directly affect transcription of target genes. These include Vascular Endothelial Growth Factor A (VegfA) leading to pancreatic vascularisation at ∼E13.5 [87]. At ∼E13.5 the pancreas begins to become vascularised leading to a negative feedback loop and the suppression of HIF target gene transcription [87,88]. High PO_2_ after E13.5 promotes proliferation of pancreatic exocrine precursors and the differentiation of endocrine cells [88–90]. Ngn3, whose expression is inversely regulated by HIF signalling, is up-regulated during this period through repression of Hes1 [88]. In adult pancreas the endocrine compartment is well vascularised and receives 10-fold the O_2_ of the surrounding exocrine pancreas [91]. The role of pO_2_ in β-cell differentiation has been explored *in vitro* using iPSCs and demonstrated to contribute to the differentiation to a β-cell phenotype including metabolic maturation (up-regulation of mitochondrial respiration) and the concurrent increases in insulin production and improved glycaemic control when transplanted to an STZ model of murine diabetes [90,92]. Together these studies suggest a key role in the induction and subsequent maintenance of endocrine function.

Given its instructive role in pancreatic development, the manipulation of O_2_ as a programming tool is historically conspicuous by its absence from nearly all liver-pancreas transdifferentiation protocols [82]. Only recently have efforts addressed the role of O_2_ in β-cell differentiation. Meivar-Levy et al. explored the idea that the vasculature may contribute to the *in vivo* maturation of the transdifferentiated β-cell phenotype. By transplanting transdifferentiated β-cells co-cultured with human bone marrow mesenchymal stem cells and endothelial colony-forming cells they found that co-cultured cells exhibited enhanced maturation marker expression, increased vascularisation of transplants and improved glycaemic control in an STZ model [93].

## Conclusion

Advances in technology, genetic engineering and cell culture techniques have greatly enhanced our ability to reprogram cells. Data from scRNA-Seq, epigenomic and proteomic projects, combined with advances in bioinformatic modelling are able to reveal, with ever greater detail, factors that may contribute to cellular reprogramming [60,62]. Improvements in the resolution with which we can interrogate cell function are challenging old assumptions while dogmas around the permanence of cellular identity have given way to a recognition of the fundamental roles epigenetics and plasticity play in determining cell identity [37]. The field of regenerative medicine continues to draw inspiration from nature to find ways to refine our ability to direct cell differentiation and transdifferentiation. By paying close attention to the environment in which β-cells exist in nature we may continue to enhance the maturation of β-cells in the laboratory. Future research should focus on pursuing this approach and consider the impact of hitherto underexplored aspects of the pancreatic environment has on β-cell identity including the roles of O_2_, the 3D environment and soluble factors.

Transcription factor-based approaches have yielded early success in the reprogramming of hepatocytes to β-cells. The present challenge is to discover how to maintain β-cells along the correct trajectory as they descend (or traverse) Waddington's landscape to their mature state [94]. A more holistic approach to cell culture, taking lessons from directed differentiation protocols, may help greatly in this. The combination of high-resolution high content technologies and faithful recapitulation of the healthy human pancreas will likely continue to yield improvements in the generation of β-cell as we edge closer to a clinically relevant therapy for T1D.

## Perspectives

- Type 1 diabetes represents a growing global healthcare burden. Current treatments fail to mitigate many of the complications. This unmet clinical need requires more effective treatment options. Transdifferentiation of liver to pancreas is one such option.
- The last 20 years has seen steady progress in the reprogramming of hepatocytes to β-cells for use as a therapy in type 1 diabetes. Improvements in our ability to recapitulate the *in vivo* environment has led to greater efficiency and functionality in generated β-cells.
- Future research should seek to even more accurately recreate the internal milieu of the pancreas in order to generate a mature cellular phenotype. Factors to be considered include the extra cellular matrix composition, oxygen tension, addition of soluble factors as well as the temporal expression of appropriate transcription factors.

## Open Access

Open access for this article was enabled by the participation of University of Bath in an all-inclusive *Read & Publish* pilot with Portland Press and the Biochemical Society under a transformative agreement with JISC.

## Abbreviations

α-cell: Alpha
ALKi: anaplastic lymphoma kinase inhibitor II
bHLH: basic helix-loop-helix
β-cell: beta
Btc: betacellulin
C/EBPβ: CCAAT-Enhancer-Binding Protein Beta
δ-cell: delta
E: embryonic day
ESC: embryonic stem cell
EGF: epidermal growth factor
ε-cells: epsilon
ECM: epidermal growth factor
FoxA2: Forkhead Box Protein A2
GATA4: GATA Binding Protein 4
ε-cell: ghrelin secreting epsilon
GSIS: glucose stimulated insulin secretion
GLUT2: glucose transporter 2
HHEX: haematopoietically expressed homeobox
HNF: hepatocyte nuclear family
HDAC: histone deacetylase inhibitor
HIF: hypoxia inducible factor
iPSC: induced pluripotent stem cell
KGF: keratinocyte growth factor
LDN: LDN193189
MafA: MAFbZIP transcription factor A
human Transferrin: human Insulin, Progesterone, Putrescine, sodium selenite, N2
Ngn3: neurogenin 3
NeuroD1: neuronal differentiation 1
OKSM: Oct4, Sox2, c-Myc and Klf4
O_2_: oxygen
PO_2_: oxygen tension
PDX1: pancreatic duodenal homeobox 1
PP-cell: pancreatic polypepetide
PCP: planar-cell-Polarity pathway
RA: retinoic acid
scRNA-seq: single cell RNA sequencing
nicotinamide: soluble vitamin B3
SCNT: somatic cell nuclear transfer
STZ: streptozotocin
TGIF2: TGFβ induced factor 2
TGFbi: TGF-β RI kinase inhibitor IV
T3: triiodo-L-thyronine
T1D: type 1 diabetes
VEGFA: vascular endothelial growth factor A
Vc: vitamin C
PCP: Wnt/planar-cell-polarity pathway
